# Targeted In-Defect Clipping After Colonic Endoscopic Resection: A Prospective Feasibility Study

**DOI:** 10.1055/a-2905-7370

**Published:** 2026-07-15

**Authors:** George Tribonias, Petros Zormpas, Marianna Spinou, Yoriaki Komeda, Antonis Pikoulas, Margarita-Eleni Manola, Eleni Nakou

**Affiliations:** 1 Gastroenterology Department 168201 General Hospital Korgialenio-Benakio Red CrossAthensAtticaGreece; 2 Gastroenterology Department 326473 Kindai University Faculty of Medicine HospitalOsaka-SayamaJapan

**Keywords:** endoscopy lower GI tract, polyps/adenomas/..., lower GI bleeding, endoscopic resection (polypectomy, ESD, EMRc, …)

## Abstract

**Background and study aims** Delayed post-polypectomy bleeding (DPPB) remains a significant adverse event following colonic endoscopic resection. Although complete defect closure may reduce bleeding risk, it is often technically challenging. We evaluated the feasibility and safety of a targeted in-defect clipping strategy that does not aim for complete closure.

**Patients and methods** We conducted a prospective feasibility cohort study of consecutive adult patients undergoing colonic endoscopic resection of lesions ≥30 mm, followed by targeted in-defect clipping (iCLIP) with 3–4 endoclips. Eligible cases involved defects in which complete closure was difficult. The primary endpoint was feasibility, defined as successful deployment of 3–4 clips in ≥90% of cases. Secondary endpoints included DPPB, delayed perforation, post-polypectomy syndrome and post-procedural pain within 30 days.

**Results** A total of 100 patients were included. Lesions were predominantly right-sided (73%) with a median size of 35 mm. EMR and ESD were performed in 71% and 29% of cases, respectively. The iCLIP strategy was successfully performed in 99% of cases, with one episode of delayed bleeding. No delayed perforation was observed. Post-polypectomy syndrome occurred in one patient and post-procedural pain in eight patients.

**Conclusions** Targeted in-defect clipping using 3–4 endoclips appears to be a feasible and safe approach for management of challenging post-resection defects and was associated with a very low rate of delayed bleeding in this pilot cohort.

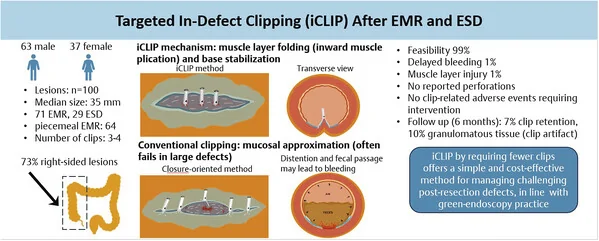

## Introduction

Endoscopic mucosal resection (EMR) and endoscopic submucosal dissection (ESD) are two well-accepted and effective techniques for managing precancerous colorectal lesions. 1 Delayed post-polypectomy bleeding (DPPB) is the most frequently encountered complication with a reported incidence of 6.5% after EMR and 1.5–11.9% after ESD. 2 3 Several risk factors have been identified including polyp size, proximal polyp location, and the use of antiplatelets/anticoagulants. 2 Despite advances in resection techniques, prophylactic complete closure of large defects can be resource-intensive and technically unfeasible. 4 5 Against this background, we evaluated a minimalist alternative approach, targeted in-defect clipping (iCLIP), aimed at stabilizing the ulcer base and exposed vessels without attempting complete closure.

## Patients and Methods



**Video 1** This video demonstrates in-defect clipping (iCLIP) procedure following four endoscopic resections of right-sided large lesions using piecemeal EMR, KAR-EMR and ESD, as well as follow-up endoscopy showing a retained clip that is removed with grasping forceps.


We conducted a prospective, feasibility cohort study of consecutive adult patients undergoing colonic endoscopic resection followed by iCLIP between October 2023 and January 2025. All procedures were carried out by a single experienced endoscopist (G.T.) at a tertiary referral center for advanced endoscopic resection. As a pilot feasibility study, no formal sample size calculation was performed; 100 consecutive patients were enrolled pragmatically to assess feasibility and safety. After lesion removal, three to four through-the-scope clips (TTSCs) were placed within the resection defect according to the study protocol. Inclusion criteria were adult patients (≥18 years) undergoing endoscopic resection of colorectal polyps ≥30 mm by either EMR (en-bloc and piecemeal) or ESD, with documented 30-day post-procedural follow-up. Antiplatelet and anticoagulant medications were managed according to ESGE guidelines. 6 Eligible patients had large mucosal defects or smaller defects located at anatomically challenging sites where complete closure was considered demanding because of factors such as colonic angulation, unstable scope position, lesion orientation, or tension across the defect. Cases requiring fewer than three clips were excluded, because complete defect closure was considered technically feasible. Rectal lesions were also excluded, because complete closure was assumed to be challenging in large lesions located in mid-lower rectum and hemostatic gel was routinely applied for bleeding prevention.

**Fig. 1:**
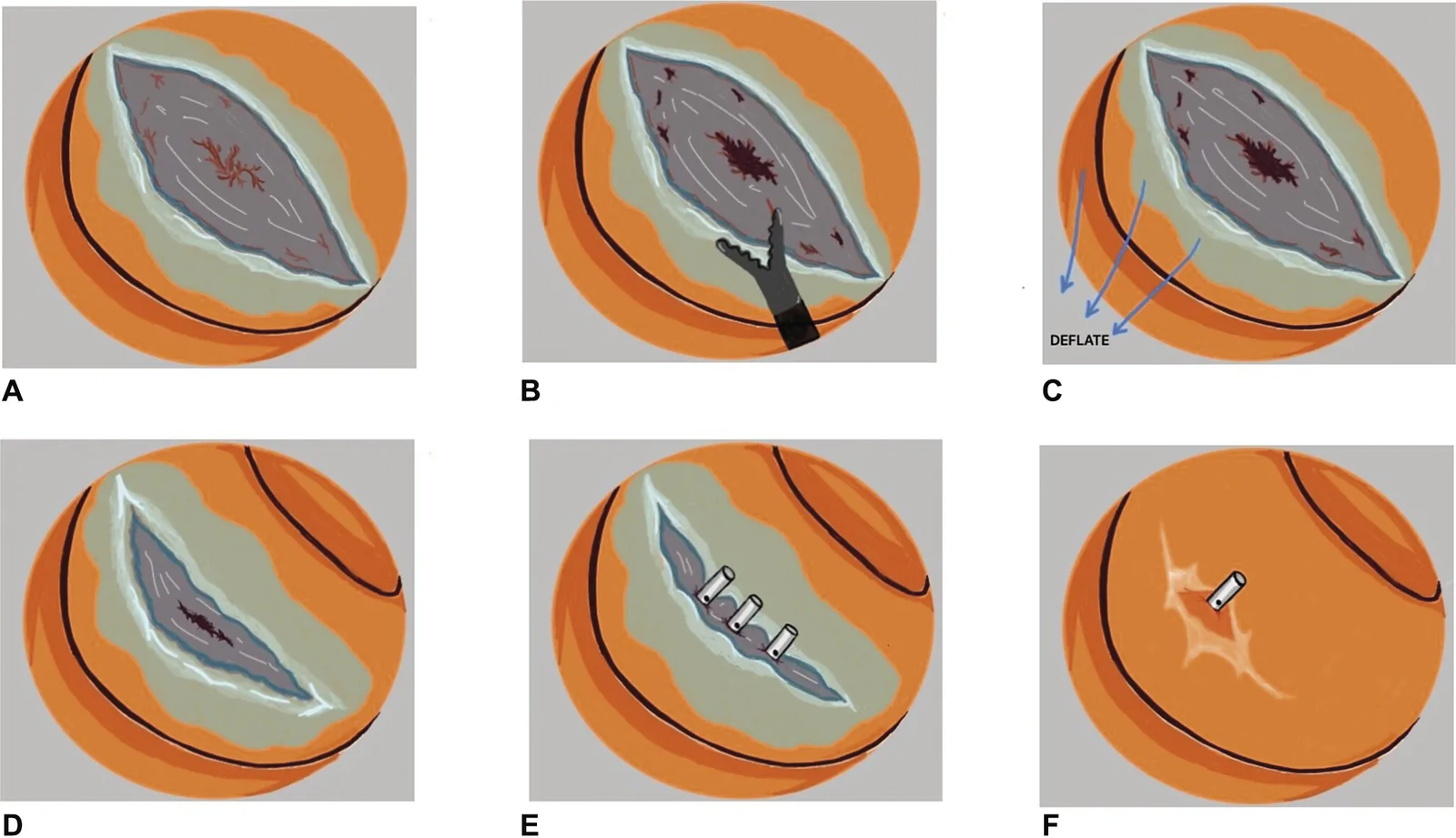
In-defect clipping technique (iCLIP). ( **A** ) Mucosal defect with large vessels at the center. ( **B** ) Soft coagulation of vessels with hemostatic forceps. ( **C** ) Deflation of the lumen enabling folding of the submucosa and muscularis propria. ( **D** ) Smaller mucosal defect. ( **E** ) In-defect clip deployment at the center without grasping the resection margins. ( **F** ) Remaining clip at the resection scar with granulomatous tissue around it.

**Fig. 2:**
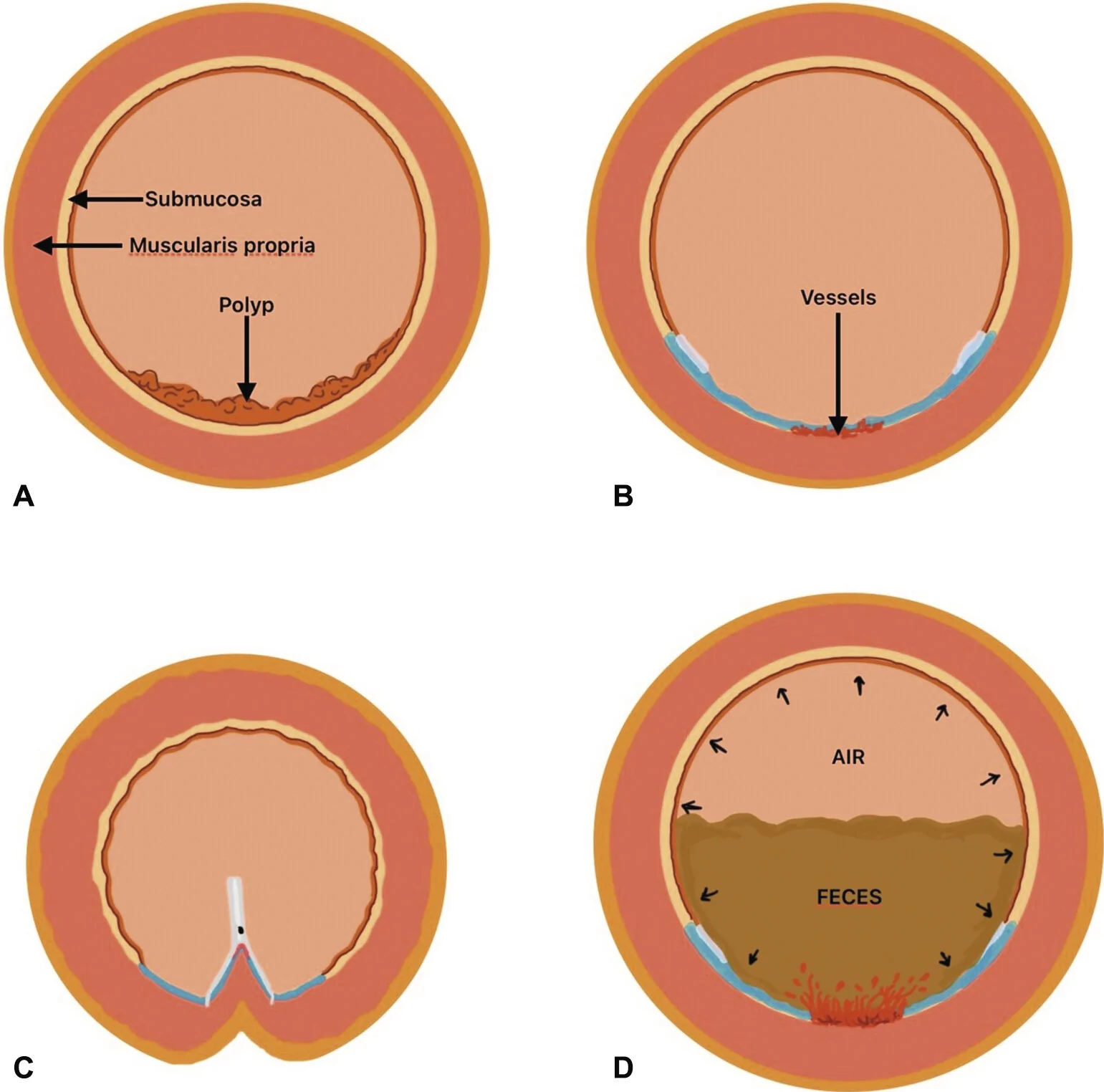
( **A** ) Polyp before endoscopic resection. ( **B** ) Mucosal defect with large vessels at the center. ( **C** ) In-defect clip placement (iCLIP). Note the folding of the muscle layer into the clip. ( **D** ) Luminal distention from gas and feces causing post-polypectomy bleeding in unclosed defect.

**Fig. 3:**
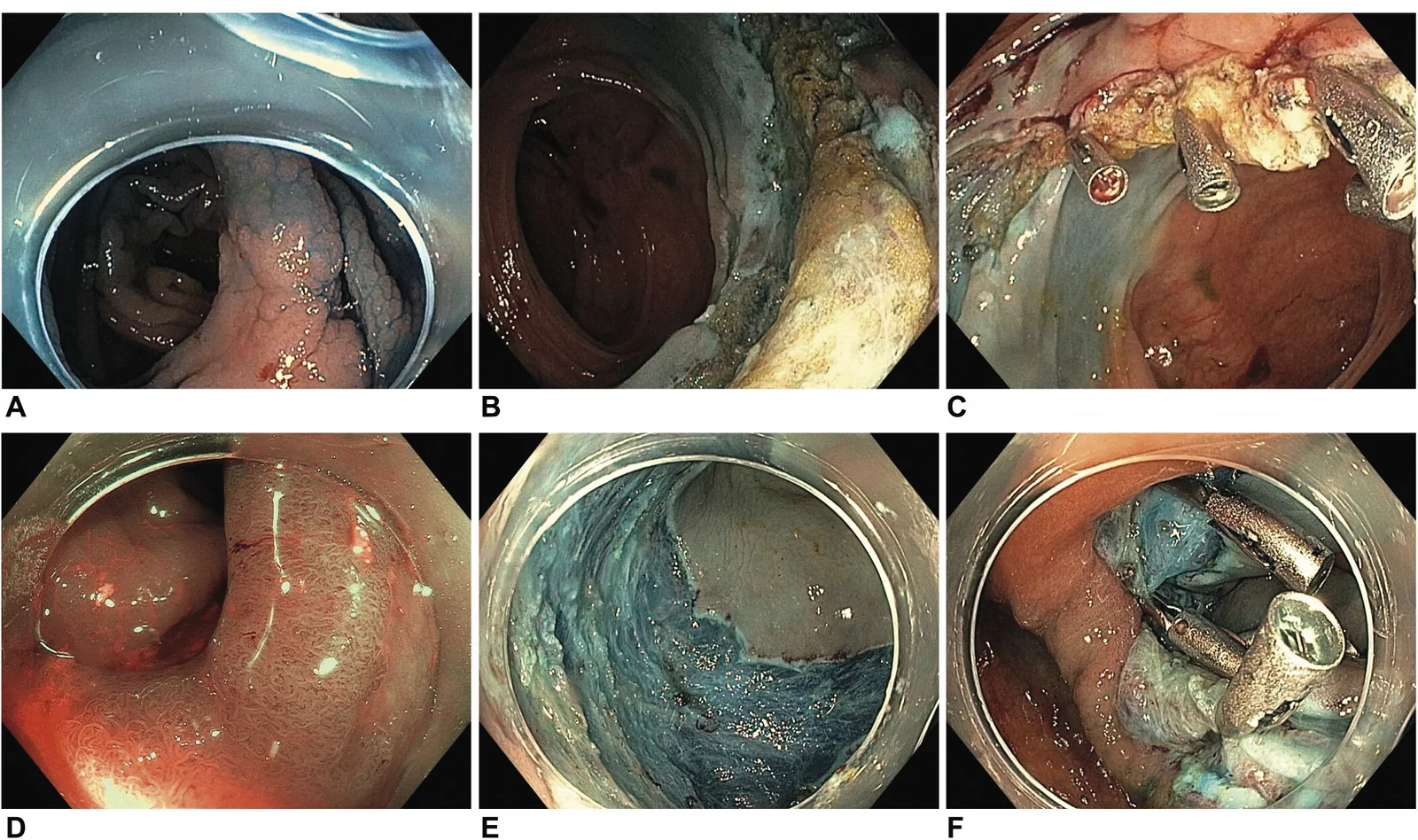
( **A** ) LST-NG flat elevated type 40 mm located at hepatic flexure. ( **B** ) Mucosal defect after ESD. ( **C** ) In-defect clipping (iCLIP) with 4 endoclips. ( **D** ) LST-NG flat elevated type 60 mm located at cecum. ( **E** ) Mucosal defect after piecemeal EMR. ( **F** ) In-defect clipping (iCLIP) with 4 endoclips.

Following completion of EMR or ESD, the resection bed was carefully inspected for exposed vessels or areas with suspected bleeding risk. All visible large submucosal vessels were prophylactically cauterized using electrocautery biopsy forceps in soft-coagulation mode. Targeted in-defect clipping was then performed using reopenable TTSCs (16-mm), with a transparent distal attachment routinely applied. Clips were deployed perpendicularly, within the ulcer base, typically beginning centrally to achieve stabilization and partial reduction of the defect. When feasible, exposed vessels were incorporated into the clipping line. Clip jaws were gently applied to the submucosal or superficial muscular layer without grasping mucosal edges. Controlled suction was applied to draw the muscularis propria into the clip before closure, resulting in localized folding of the muscle layer inward ( Figs. 1 and 2 ). Excessive pressure was deliberately avoided to minimize the risk of mural injury or perforation. Areas with visible deep mural injury or markedly thinned muscularis propria were deliberately avoided whenever possible. Additional clips were placed adjacently using the same technique, without attempting complete closure ( Fig. 3 , Video 1 ).

ESD patients were discharged the next day and EMR patients the same day. Antibiotics were not routinely administered. Patients were monitored for 30 days for adverse events and underwent surveillance endoscopy at 6 months, with extended follow-up when clinically indicated.

The primary endpoint was feasibility, defined as successful deployment of 3–4 endoclips for in-defect clipping in ≥90% of eligible cases. Secondary endpoints included delayed bleeding and delayed perforation occurring within 30 days after resection.

Additional secondary outcomes were post-polypectomy syndrome (localized peritoneal signs with systemic inflammation 12 h–5 days after procedure, without radiologic perforation) and post-procedural pain (abdominal pain within 24 h after polypectomy without systemic inflammation).

Data on patient and lesion characteristics, as well as the endoscopic resection technique used, were prospectively collected. Recorded variables included age, sex, use of antiplatelet or anticoagulant therapy, polyp morphology (according to Paris and JNET classification), location, size, and histopathological diagnosis. Additional data related to the iCLIP procedure included number of clips used per case, clip-related perforation or muscle layer injury, duration of the procedure, and clinically significant clip migration. Follow-up endoscopy assessed local recurrence, clip-related artifacts, and retained clips.

The study was conducted according to the Declaration of Helsinki (2013) and Good Clinical Practice (European Guidelines from 1997). Institutional review board approval was obtained (registration number: 334/20703).

### Statistical Analysis

Descriptive statistics were used. Continuous variables are presented as medians with interquartile ranges and categorical variables as frequencies and percentages. Analyses were performed using Microsoft Excel.

## Results

A total of 100 patients were included. Twenty-four patients were receiving antithrombotic therapy. Lesions were predominantly right-sided (73%) with a median size of 35 mm. EMR and ESD were performed in 71% and 29% of cases, respectively. Among the EMR cases, the majority involved piecemeal resection (64%). No full-thickness mural injuries or perforations caused by EMR or ESD were identified before clip deployment. Median total iCLIP procedure time was 222 seconds, while median individual clip deployment time was 17 seconds. Histology revealed adenoma (71%), sessile serrated lesions (SSLs) (21%), adenocarcinoma (5%), and hyperplastic polyps (3%). In-defect clipping was successfully performed in 99% of cases. One episode of delayed post-procedural bleeding was observed. No delayed perforation or clinically significant clip migration was demonstrated. Post-polypectomy syndrome occurred in one patient and post-procedural pain in eight. One localized muscularis propria injury occurred during clip placement without evidence of true perforation ( Table 1 ). At surveillance endoscopy, small (5-mm) local recurrence was identified in two patients. Granulomatous tissue was observed in ten patients and a retained clip in seven ( Table 1 , Fig. 1 ).

**Table 1:** Descriptive statistics.

Variable	*N* = 100
**Age, Median (IQR)**	68 (62–75)
**Size, Median (IQR)**	35 (30–40)
** Clips, *n* (%) **	
3	62
4	38
** Resection method, *n* (%) **	
EMR (piecemeal)	64
EMR (en-bloc)	7
ESD	29
** Sex, *n* (%) **	
Female	37
Male	63
** Location, *n* (%) **	
Ascending	42
Cecum	17
Descending	7
Sigmoid	20
Transverse	14
** Antiplatelets/Anticoagulants, *n* (%) **	24
Aspirin	12
P2Y12 inhibitors	3
DOACs	8
Warfarin	1
** Paris classification, *n* (%) **	
0-IIa	47
0-IIa+c	4
0-IIa+Is	18
Ip	5
Is	13
Isp	12
Subepithelial	1
** JNET, *n* (%) **	
1	16
2A	59
2B	25
** Histology, *n* (%) **	
Adenoma	71
Carcinoma	5
Hyperplastic	3
SSL	21
** Technical success, *n* (%) **	99
**Total Procedure time (s), Median, (IQR)**	222 (182–258)
**Individual clip placement time (s), Median, (IQR)**	17 (14–26)
** Delayed bleeding, *n* (%) **	1
** Delayed perforation, *n* (%) **	0
** Muscle layer injury, *n* (%) **	1
** Clip induced perforation, *n* (%) **	0
** Clinically significant clip migration, *n* (%) **	0
** Post-polypectomy syndrome, *n* (%) **	1
** Post-procedural pain, *n* (%) **	8
** Recurrence, *n* (%) **	2
** Remaining clip, *n* (%) **	7
** Clip artifact (granulomatous tissue), *n* (%) **	10

## Discussion

In this pilot cohort, iCLIP following colonic EMR or ESD was highly feasible and associated with near-complete absence of delayed bleeding. The single delayed bleeding event resolved spontaneously without intervention or blood transfusion; notably, vertical clip deployment was unfeasible in that case due to acute angulation. No delayed perforations were recorded. A single localized muscularis propria injury occurred during clip deployment following ESD, without visible extraluminal fat, target sign, or evidence of full-thickness perforation; the patient remained asymptomatic and required no therapeutic intervention. Rates of post-polypectomy syndrome and post-procedural pain were comparable to those reported after colonic endoscopic resection. 7

Because iCLIP was also applied after piecemeal EMR, a theoretical concern is that inward tissue plication could affect scar appearance or recurrence detection. In this cohort, however, recurrent lesions were small (5 mm), readily identifiable at surveillance endoscopy and successfully treated endoscopically. Retained clips were removed uneventfully and clip-related granulomatous tissue showed no dysplasia on histology. The majority of them had completely resolved by the 18-month follow-up endoscopy.

The potential mechanism of iCLIP may involve stabilization of the ulcer base and exposed vessels through localized inward tissue plication, potentially reducing mechanical stress during the early post-resection period. This concept may be particularly relevant in the right colon, where luminal distension, peristalsis, and exposure to liquid stool have been proposed as contributing factors to delayed bleeding after large-polyp resection 2 8 9 ( Fig. 2 ). Because clinically significant exposed vessels are frequently encountered in the central resection defect, targeted clipping of these areas may theoretically enhance hemostatic stability even in the absence of complete defect closure. Nonetheless, these proposed mechanisms remain speculative and require further evaluation.

Muscle-layer folding has previously been described in the “Origami” method. 5 However, unlike “Origami,” which aims for complete defect closure, iCLIP uses limited in-defect clipping to stabilize the ulcer base and exposed vessels without full mucosal approximation. This may simplify the technique and extend its applicability to defects not amenable to complete closure.

Randomized trials support prophylactic clipping after EMR of right-sided large non-pedunculated polyps, with bleeding reduction observed only after complete closure. 9 10 In the CLIPPER trial, complete closure was achieved in 72% of cases, while incomplete closure was associated with significantly higher delayed bleeding. 10 The reported incidence of delayed bleeding is 6.5% after EMR and 1.5–11.9% after ESD. 2 3 9 In this pilot cohort, the delayed bleeding rate was 1% despite the predominance of right-sided lesions. Although the mechanism remains uncertain, iCLIP may provide more durable stabilization of the post-resection defect. Clip retention in a subset of cases may reflect more stable clip anchoring within the defect than mucosal edge approximation alone. Moreover, this technique directly compresses submucosal and muscular vessels, which are primarily responsible for delayed bleeding. However, this observation remains descriptive.

Delayed post-polypectomy bleeding has been reported more frequently after EMR than after ESD, suggesting technique-related differences in bleeding risk. 1 2 3 Our cohort included both EMR (en-bloc and piecemeal) and ESD cases, but with a higher rate of piecemeal resections. Proximal lesion location is a well-established independent risk factor for delayed bleeding. 2 9 10 Our cohort included 73% of right-sided lesions and 64% piecemeal EMR resections, a combination that represents the highest-risk population for delayed bleeding according to the current literature. 2 9 10

In cases of piecemeal EMR, snare-tip soft coagulation was not routinely used. Expansion of the mucosal defect was performed with snare polypectomy rather than STSC. However, prophylactic coagulation of visible vessels was performed before clip placement as part of the study protocol, because of large post-resection defects. Therefore, the independent contribution of iCLIP to the observed outcomes should be interpreted with caution.

Antithrombotic therapy is a recognized risk factor for DPPB. 2 In this cohort, 24% of patients were receiving antiplatelet or anticoagulant therapy and managed according to ESGE recommendations. 6 The single delayed bleeding event occurred after resumption of a direct oral anticoagulant (DOAC). Although conclusions cannot be drawn from a non-comparative study, the very low observed rate of delayed bleeding despite the inclusion of higher-risk patients is of interest.

Approximately 20% of lesions were SSLs. Although non-dysplastic SSLs may be suitable for cold resection, which is associated with lower rates of DPPB, all lesions in this cohort were ≥30 mm and showed endoscopic features suggestive of dysplasia, warranting electrocautery-based resection.

Randomized trials evaluating delayed bleeding have focused on large colorectal lesions measuring ≥20 mm, with lesion size ≥40 mm consistently identified as a major risk factor for delayed bleeding. 2 7 In the present cohort, the median lesion size was 35 mm; however, larger defects can be managed using the same iCLIP technique with additional clips as needed.

Median total iCLIP procedure time was 3.42 min. Although no direct comparison was performed, iCLIP may be less time-consuming than conventional clipping, because it does not require mucosal edge approximation or complete closure. Moreover, reduction in number of clips and avoidance of specialized closure devices support principles of cost-consciousness and green endoscopy. Although the present study was not designed to assess economic or environmental outcomes, these considerations warrant further evaluation.

Despite limitations including a single-center design, lack of a control group, and performance by a single expert operator, the low incidence of DPPB is encouraging and supports further evaluation in comparative prospective studies.

In conclusion, iCLIP using three to four clips appears to be a feasible and safe alternative to full defect closure after large colonic EMR or ESD. The very low observed delayed bleeding rate supports further evaluation in comparative prospective studies.

## References

[1] Hassan C Repici A Sharma P Efficacy and safety of endoscopic resection of large colorectal polyps: a systematic review and meta-analysis Gut 2016 65 05 806 820 25681402 10.1136/gutjnl-2014-308481

[2] Burgess N G Metz A J Williams S J Risk factors for intraprocedural and clinically significant delayed bleeding after wide-field endoscopic mucosal resection of large colonic lesions Clin Gastroenterol Hepatol 2014 12 04 651.e1-3 61.e1-3 24090728 10.1016/j.cgh.2013.09.049

[3] Miyakawa A Tamaru Y Mizumoto T Prophylactic clip closure after endoscopic submucosal dissection of large flat and sessile colorectal polyps: a multicentre randomised controlled trial (EPOC trial) Gut 2025 74 11 1814 1820 40461060 10.1136/gutjnl-2024-334463 PMC12573426

[4] Gong R Wang S Song J Closure methods for large defects after gastrointestinal endoscopic submucosal dissection J Gastroenterol Hepatol 2024 39 12 2511 2521 39175260 10.1111/jgh.16722 PMC11660212

[5] Masunaga T Kato M Sasaki M Modified double-layered suturing for a mucosal defect after colorectal endoscopic submucosal dissection (origami method) (with video) Gastrointest Endosc 2023 97 05 962 969 36642200 10.1016/j.gie.2023.01.005

[6] Veitch A M Radaelli F Alikhan R Endoscopy in patients on antiplatelet or anticoagulant therapy: British Society of Gastroenterology (BSG) and European Society of Gastrointestinal Endoscopy (ESGE) guideline update Gut 2021 70 09 1611 1628 34362780 10.1136/gutjnl-2021-325184 PMC8355884

[7] Mann R Gajendran M Umapathy C Endoscopic Management of Complex Colorectal Polyps: current insights and future trends Front Meds 2022 20 08 728704 10.3389/fmed.2021.728704 PMC8811151 35127735

[8] Bendall O James J Pawlak K M Delayed bleeding after endoscopic resection of colorectal polyps: identifying high-risk patients Clin Exp Gastroenterol 2021 24 14 477 492 10.2147/CEG.S282699 PMC8714413 34992406

[9] Spadaccini M Albéniz E Pohl H Prophylactic clipping after colorectal endoscopic resection prevents bleeding of large, proximal polyps: meta-analysis of randomized trials Gastroenterology 2020 159 01 148 1.58E13 32247023 10.1053/j.gastro.2020.03.051

[10] Kemper G Turan A S Schreuder R M The effect of prophylactic clipping on delayed bleeding after proximal colonic endoscopic mucosal resection: a multicenter, randomized controlled trial (CLIPPER) Endoscopy 2025 57 11 1243 1250 40695477 10.1055/a-2637-3180

